# Developmental exposure to sertraline impaired zebrafish behavioral and neurochemical profiles

**DOI:** 10.3389/fphys.2022.1040598

**Published:** 2022-11-18

**Authors:** Melissa Faria, Marina Bellot, Oscar Soto, Eva Prats, Nicola Montemurro, Diana Manjarrés, Cristian Gómez-Canela, Demetrio Raldúa

**Affiliations:** ^1^ Institute for Environmental Assessment and Water Research (IDAEA-CSIC), Barcelona, Spain; ^2^ Department of Analytical and Applied Chemistry (Chromatography Section), School of Engineering, Institut Químic de Sarrià-Universitat Ramon Llull, Barcelona, Spain; ^3^ Universitat Ramon Llull, Barcelona, Spain; ^4^ Research and Development Center (CID-CSIC), Barcelona, Spain

**Keywords:** sertraline, zebrafish larvae, neurotransmitters, development, environmental concentrations

## Abstract

The number of people suffering from mental health problems is rising, with anxiety and depression now the most commonly diagnosed psychiatric conditions. Selective serotonin reuptake inhibitors (SSRIs) are one of the most prescribed pharmaceuticals to treat these conditions, which has led to their common detection in many aquatic ecosystems. As the monoaminergic system shows a high degree of structural conservation across diverse animal phyla, a reasonable assumption is that the environmental levels of SSRIs in surface water can lead to adverse effects on fish and other aquatic wildlife. For instance, Sertraline (SER), a widely prescribed SSRI, has been shown to induce adverse effects in fish, albeit most of the reports used exposure concentrations exceeding those occurring in natural environments. Therefore, there is still a great lack of knowledge regarding SERs effects in fish species, especially during early life stages. This study describes the evaluation of developmental exposure of zebrafish (*Danio rerio*) to environmentally relevant concentrations of SER (from 0.01 to 10 μg/L), using a battery of key survival behaviors and further relating them with the expression of genes and neurochemical profiles of the monoaminergic system. We found that developmental exposure to SER did not affect embryo morphogenesis and growth. However, concentrations as low as 0.1 μg/L induced hypolocomotion and delayed learning. The observed behavioral impairment was associated with augmented serotonin levels rather than other neurochemicals and molecular markers, highlighting the relationship between serotonin signaling and behavior in zebrafish.

## 1 Introduction

Antidepressants support millions of people around the world. The majority of the OECD (Organization for Economic Cooperation and Development) countries showed an increasing trend in their consumption since 2000, with Iceland reporting the most significant increase, which has doubled the average of all OECD countries, followed by Australia, Portugal and Canada (Sehonova et al., 2018). Furthermore, after the entry into force of the social measures linked to the COVID pandemic, a considerable increase on the prescription of these drugs has been detected (Diaz-Camal et al., 2022). Amongst one of the most prescribed class of antidepressants are the selective serotonin reuptake inhibitors (SSRIs) (Macaluso and Preskorn, 2019), which block the reuptake of serotonin at the pre-synaptic terminals for enhancing serotonin signaling in the post-synaptic neuron (Gornik et al., 2020). Currently six SSRIs are commercially available: citalopram, escitalopram, fluoxetine, fluvoxamine, paroxetine and sertraline. After their administration, SSRIs can be excreted directly or after undergoing metabolic biotransformation, finally reaching wastewater treatment plants (WWTPs). It has been reported that metabolites of these drugs can cause similar effects as their parent compound (Huang et al., 2019). As a result of the poor removal efficiency of many WWTPs, SSRIs are commonly detected in most of the aquatic ecosystems worldwide (Gornik et al., 2020; de Souza et al., 2021; Miller et al., 2021). The presence of SSRIs in aquatic ecosystems has raised a concern of unknown possible toxic effects they may cause in aquatic biota. Of all SSRIs, sertraline (SER) is one of the most frequently prescribed (Top 25 Psychiatric Medications for 2018) and consequently has also become one of the most frequently detected in surface water and WWTP effluents (Mole and Brooks, 2019). The concentration of SER in surface water can be found in the ng/L to µg/L range, and has been shown to induce adverse effects, especially in fish species inhabiting affected areas (Schultz et al., 2011) (Valenti et al., 2012) (Hedgespeth et al., 2014). Sertraline is fairly lipophilic (logK_ow_ = 5.29), so it passively diffuses through lipid membranes and is therefore known to accumulate in high lipid content tissues including the brain (Xie et al., 2017; Hubená et al., 2020). As the serotonin system is phylogenetically well conserved between fish and mammals (Panula et al., 2006), fish continuously exposed to environmental levels of SSRIs, such as, SER could result in physiological/behavioral changes (Kreke and Dietrich, 2008). In the wild, even small changes in specific behaviors, are likely to have ecological repercussions that in turn could affect the population. Indeed, fish exposure to SER during different lengths of time as well as during different developmental stages has been demonstrated to alter fish behavior (Xie et al., 2017; Suryanto et al., 2021; Yang et al., 2021). For example, Xie et al., 2017 observed decreased shoaling tendency and feeding rate and increased swimming activity in juvenile crucian carp. In zebrafish, Yang et al., 2021 observed significant reduction in 6 days post fertilization) (dpf) zebrafish larvae locomotor activity, under dark conditions, following developmental exposure to 1–100 μg/L of SER while Suryanto e al., 2021 observed, in 5dpf embryos an increase in locomotor activity during the light cycle following only 24 h exposure to 1 mg/L. However, despite the existing information, there is still much unknown regarding SER effect in fish species, especially during early life stages. It is widely accepted that the developing brain is more sensitive to chemical exposure than the adult brain (Giordano and Costa, 2012), and exposure to neuroactive chemicals during this sensitive stage may cause permanent changes to the central nervous system (CNS). This study aims to further contribute to narrowing this gap of knowledge, by measuring neurobehavioral effects in zebrafish embryos/larvae following exposure to environmentally relevant concentrations of SER during embryonic and early larvae developmental stages. The selected endpoints include four different larval behaviors, the expression of genes playing a key role in the monoaminergic system as well as the levels of neurochemicals related to the monoaminergic system.

## 2 Materials and methods

### 2.1 Fish husbandry and larvae production

Adult wild-type zebrafish were obtained from Exopet (Madrid, Spain) and maintained in fish water [reverse-osmosis purified water containing 90 μg/ml of Instant Ocean (Aquarium Systems, Sarrebourg, France) and 0.58 mM CaSO_4_.2H_2_O] at 28 ± 1°C in the Research and Development Centre of the Spanish Research Council (CID-CSIC) facilities under standard conditions. Embryos were obtained by natural mating, collected using a mesh strainer and maintained in renewed fish water (pH 7.8 ± 0.2 and a conductivity of 800 ± 55 µS) in a thermostatic chamber (POL-EKO APARATURA Climatic chamber KK350, Poland) at 28.5°C on a 12L:12D photoperiod at one embryo/mL ratio. Zebrafish embryos under these conditions begin hatching from 48 to 72hpf, after which they are considered larvae (Kimmel et al., 1995). Embryo/larvae were not fed during the experimental period to avoid interference with exposure concentrations of SER. All procedures were approved by the Institutional Animal Care and Use Committees at the CID-CSIC and conducted following the institutional guidelines under a license from the local government (agreement number 11336).

### 2.2 Experimental concentrations and stability of SER in fish water

Sertraline Hydrochloride (CAS: 79559-97-0, ≥98%) (SER) was obtained from (Merck, United States ). Experimental concentrations in fish water of the four nominal concentrations used in this study (0.01 μg/L, 0.1 μg/L, 1 μg/L, and 10 μg/L), with three replicates for each concentration, were determined using ultra-high-performance liquid chromatography with triple quadrupole detector (UHPLC-MS/MS). Furthermore, the stability of SER in fish water was assessed by preparing triplicate solutions of the four selected concentrations, which were maintained under the exposure conditions (28 °C and 12L:12D photoperiod). Aliquots of these solutions were analyzed at times 0, 24, and 48 h.

### 2.3 Experimental protocol

Stock solutions of SER were prepared in dimethyl sulfoxide (DMSO), and then diluted in fish water to a final carrier concentration of 0.1% in all treatments. The four selected SER concentrations are within the range of those found in surface waters and those detected in fish plasma (Valenti et al., 2012).

Two independent experiments were carried out. All exposures were performed at 28.5°C (POL-EKO APARATURA Climatic chamber KK350, Poland) with 12L:12D photoperiod. For each assay, samples for each analysis were collected from at least two independent experiments. Zebrafish embryos were previously sorted to the same developmental of the blastula period stage (approximately the oblong and sphere stage). Following this, embryos were then exposed to four environmental relevant concentrations of SER (0.01, 0.1, 1, and 10 μg/L) from four *hpf* (hours post fertilization) to eight *dpf* (days post fertilization). Exposure was conducted in 6-well plates with 10 embryos per well containing 10 ml of medium, and a total of 12 wells for each treatment. The medium was changed every 48 h. Survival and hatching were recorded every 24 h starting from the beginning of the exposure, according to the OECD 212, “Test Guideline 212”, 1998 (Test No. 212: Fish, Short-term Toxicity Test on Embryo and Sac-Fry Stages, 1998) standards, along with any obvious morphological abnormalities. Total body length and truck thickness was measured in 12–20 larvae at the end of the exposure (8 dpf). Larvae selected for behavioral tests were transferred 24 h before trials to 48-well plates where one larva was placed in each well containing 1 ml of exposure medium.

### 2.4 Fixation and morphometric measurements

Zebrafish larvae were collected and fixed overnight at 4 °C in phosphate buffered saline 1× solution (PBS) with 4% PFA (paraformaldehyde) as previously described (Garcia-Reyero et al., 2014). For detailed morphological observation and larvae measurements, fixed larvae were then washed several times with PBS and gradually transferred to 90% glycerol in PBS for long-term preservation and positioning under the stereomicroscope.

All individuals were observed and photographed under a Nikon SMZ1500 (NIKON Instruments INC. New York, United States ) dissecting microscope fitted with Nikon Digital Sight DSRi1 camera and NIS Elements AR software (version 3.0) (NIKON Instruments INC. New York, United States ) and saved as high resolution (3840 × 3005 pixels) tagged image file format (TIFF). All images were taken on the left side of each larva. The total body length (anterior-most part of the snout to posterior-most point of the tail, mm) and trunk thickness (mm) were obtained using a measuring tool from the NIS Elements software.

### 2.5 Behavioral analysis

Experiments were conducted in 48-well plates at one larva per well, maintained under dark before transferring them to a behavioral testing chamber equipped with a temperature control unit set to 28°C (DanioVision, Noldus Information Technology, Leesburg, VA). Once inside the chamber, larvae were left to acclimate in the dark for a further 10 min before initiating video recording.

The vibrational startle response assay was performed under near-infrared light as described in Faria et al., 2019) (*n* = 79–82). 50 consecutive tapping stimuli selected at the highest intensity (intensity level: 8) were delivered every second. Videos were recorded at 30 frames per second and the vibrational startle response (VSR or Startle) was analyzed for each larva by leveling basal movement to Y = 0 and then measuring the distance moved (cm) over the 1 s period following the first stimulus, while the habituation of the startle responses was measured as the area under the curve (AUC) of plots of distance moved relative to the response of the first stimulus (n = 76–82). Video tracking and measurement of the startle response were analyzed using the EthoVision XT 13 software (Noldus, Wageningen, Netherlands).

Basal locomotor activity (BLM) and visual motor response (VMR) analyses of eight dpf zebrafish larvae were performed also using a DanioVision system running an Ethovision XT 13 software essentially as described by Faria et al., 2015). The BLM (*n* = 76–83), represents the total distance (cm) traveled by each larva during 10min under dark conditions, while the VMR (*n* = 68–84) reports larvae responses following a transition of light to dark and is represented as the difference of the total distance (cm) traveled during 2 minutes after and before the transition. Video tracking conditions consisted of a 40 min cycle including a 15 min dark period, followed by a 10 min light period and then a second 15 min dark cycle. The position of each larva was recorded using an IR digital video camera Basler acA1300-60 g m (Basler Inc., Exton, PA) and an EthoVision XT 13 video tracking system. All measurements were performed between 10:00 and 16:00 h. Tracks were analyzed in terms of total distance moved (cm) calculated for each dark or light period. All microplates were analyzed at 28 ± 0.5 °C with the same detection and acquisition settings.

### 2.6 RNA preparation and qRT-PCR analysis

Gene expression analysis was performed as previously reported (Prats et al., 2017). Total RNA was extracted from a total of six pools of four larvae (8 dpf) using the Trizol Reagent (Invitrogen Life Technologies, Carlsbad, CA). RNA concentration was measured by spectrophotometric absorption at 260 nm in a NanoDrop™ ND-8000 spectrophotometer (Fisher Scientific) and the quality was checked in an Agilent 2100 Bioanalyzer (Agilent Technologies, Santa Clara, CA). RIN (RNA Integrity Number) values ranged between 9 and 10. After DNase I treatment (Ambion, Austin, TX), 1 µg of total RNA was used to synthesize the first strand of cDNA with the First Strand cDNA synthesis Kit (Roche Diagnostics, Mannheim, Germany) using oligo (dT), following the manufacturer’s instructions.

Real-Time PCR was performed in a LightCycler®480 Realtime PCR System using SYBR Green PCR Master Mix (Roche Diagnostics, Mannheim, Germany). Cycling parameters were 95°C for 15 min followed by 45 cycles of 95°C, 10 s and 60°C, 30 s. For each experimental condition, qPCR analyses were performed from two independent experiments, with six biological replicates on each experiment and three technical replicates for each sample. Primer sequences of the seven selected genes related to the monoaminergic system (*tph1a*, *sert (or slc6a4a)* and *vmat2 (or slc18a2)*, *dat* (or *slc6a3*), *dbh*, *th1* and *th2*) are reported in Table 1. The housekeeping gene *ppiaa* was used as a reference gene for normalization purposes (Prats et al., 2017). Primers were synthesized by Sigma-Aldrich (Steinheim, Germany). The efficiency and specificity of all primers were checked before the analyses. Results were normalized to *ppiaa* and the relative abundance of mRNA was calculated following the *ΔΔ*Ct method (Livak and Schmittgen, 2001) deriving fold-change ratios from these values.

**TABLE 1:** List of primers used for qPCR.

Gene (description)	ZFIN Acc number	GenBank Acc number		Sequence	Amplicon length
*tph1a* (tryptophan hydroxylase 1*a*)	ZDB-GENE-030317-1	NM_178306.3	FW	5′-CAG​TTC​AGT​CAG​GAG​ATT​GG	176 bp
			RV	5′-GAC​AGT​GCG​TGC​TTC​AG	
*sert* (sodium-dependent serotonin transporter *slc6a4a*)	ZDB-GENE-060314-1	NM_001039972.1	FW	5′-TAA​CCA​CTA​CAG​TTT​GGC​TTG​ATG	147 bp
			RV	5′-AAC​AGT​TAA​CCG​AGC​TTG​TGA​T	
*vmat2* (vesicular monoamine transporter 2 *slc18a*2)	ZDB-GENE-080514-1	NM_001256225.2	FW	5′-TGG​AGC​TCT​GCA​GCT​TTT​TGT​GC	159bp
			RV	5′-AAC​GCC​GGC​TCC​AGC​ATA​GC	
*dbh* (dopamine-β-hydroxylase)	ZDB-GENE-990621-3	NM_001109694	FW	5′-TGC​AAC​CAG​TCC​ACA​GCG​CA	156 bp
			RV	5′-GCT​GTC​CGC​TCG​CAC​CTC​TG	
*dat* (dopamine transporter *slc6a3*)	ZDB-GENE-010316-1	NM_131755	FW	5′-AGA​CAT​CTG​GGA​AGG​TGG​TG	151 bp
			RV	5′-ACC​TGA​GCA​TCA​TAC​AGG​CG	
*th1* (tyrosine hydroxylase 1)	ZDB-GENE-990621-5	NM_131149.1	FW	5′-GAC​GGA​AGA​TGA​TCG​GAG​ACA	95 bp
			RV	5′-CCG​CCA​TGT​TCC​GAT​TTC​T	
*th2* (tyrosine hydroxylase 2)	ZDB-GENE-050201-1	NM_001001829.1	FW	5′-CTC​CAG​AAG​AGA​ATG​CCA​CAT​G	110 bp
			RV	5′-ACG​TTC​ACT​CTC​CAG​CTG​AGT​G	
*ppiaa* (2-peptidylprolyl isomerase A)	ZDB-GENE-030131-8556	NM_212758.1	FW	5′-GGG​TGG​TAA​TGG​AGC​TGA​GA	179 bp
			RV	5′-AAT​GGA​CTT​GCC​ACC​AGT​TC	

### 2.7 Extraction and neurotransmitters analysis

Crystalline solid standards and internal labeled standards (IS) of the selected monoaminergic neurochemicals (Table 2) were obtained from Toronto Research Chemicals (TRC, Toronto, Canada), Merck (Darmstadt, Germany), and Sigma-Aldrich (St. Louis, MO, United States ), detailed information can be found in a previous study (Mayol-Cabré et al., 2020). Monoaminergic neurochemicals were extracted from four to six pools of 15 larvae heads following an extraction procedure adapted from a previous study (Bellot et al., 2021). Briefly, larvae were euthanized by chilling prior to decapitation, larvae were then positioned on the lateral side under a dissecting microscope. Heads were sectioned immediately from caudal to the otic vesicle and cranial to the anterior intestine, pooled and collected into a 1.5 ml tube. The excess medium was removed and heads were immediately frozen in dry ice and stored at -80°C. The extraction process was based on the use of a solvent of polarity similar enough to the neurotransmitters to be able to extract them from the sample. Next, 300 μL of cold extractant solvent (ACN:H_2_O (90:10) + 1% formic acid) were added to pools of larvae heads. Each sample was spiked with 50 ng of an internal standard mixture (ISM), except for 5-HT-d_4_ which was spiked at 20 ng. Three stainless steel beads (3 mm diameter) were placed in each pool and samples were homogenized using a bead mill homogenizer (TissueLyser LT, Quiagen, Hilden, Germany) at 50 osc/s for 90s and centrifuged for 20 min at 15870 g at 4°C. Finally, the supernatant was filtered using 0.20 μm PTFE filters (DISMIC -13 JP, Advantec^®^) and kept at -80°C until the LC-MS/MS analysis. Temperature is a key element throughout the extraction process because neurotransmitters are very unstable and high temperatures can degrade these compounds. As commented before, the analysis was performed by ultra-high-performance liquid chromatography (Acquity UPLC H-Class Waters, Milford, MA, United States ) coupled to a triple quadrupole mass spectrometer equipped with an electrospray (ESI) source (Xevo TQ-S micro, Waters, United States ) (Mayol-Cabré et al., 2020; Bellot et al., 2021). Neurotransmitter levels were then normalized by total protein determined using Bradford Method (Bradford, 1976), and represented as ng/mg of protein.

**TABLE 2:** Analyzed monoaminergic and catecholaminergic neurochemicals.

Neurochemical	Description
serotonin (5-HT)	Monoamine neurotransmitter
5-hydroxyindoleacetic acid (5-HIAA)	5-HT metabolite by MAO# activity
norepinephrine	Catecholamine neurotransmitter synthesized from dopamine
Dopamine	Catecholamine neurotransmitter
3,4- dihydroxyphenylacetic acid (DOPAC)	Dopamine metabolite through MAO# activity
3-methoxytyramine (3-MT)	Dopamine metabolite through COMT* activity
Homovanillic acid (HVA)	Dopamine major metabolite through consecutive action of MAO and COMT
Tyrosine	Dopamine precursor
Levodopa (l-DOPA)	Dopamine and norepinephrine precursor

# monoamine oxidase *catechol-O-methyltransferase enzyme

### 2.8 Chemical analysis of SER and its stability during 48 hours

Highly pure (>99%) SER analytical standard and sertraline-d_3_ internal standard were used to define its stability. Both standards (native and labeled) were purchased from Sigma-Aldrich (St Louis, Missouri, U.S.). Stock solution of SER was prepared in 100% dimethyl sulfoxide (DMSO, Merck Darmstadt, Germany). Individual IS was directly obtained as a solution (1 mg/ml) in acetonitrile (ACN). Work solution of IS was prepared at a concentration of 2 mg/L. For the chromatographic separation and the MS analysis, high purity mobile phase solutions were prepared using ACN and HPLC water (Optima™ LC-MS Grade) purchased from Fisher Chemical (Fisher Scientific SL, Madrid, Spain). For SER stability, serial dilutions were prepared from a stock of 1490 mg/L. The spiked fish water was prepared with SER concentrations from 0.01 ng/ml to 10 ng/ml and its stability was evaluated at 0, 24, and 48 h in triplicate. Aliquots of 1 ml of spiked water were added to a 2-ml vial in which 5 µL of 2 mg/L sertraline-d_3_ solution in ACN were previously evaporated (Final IS concentration: 10 ng/ml). Vials were accurately vortexed for 2 min using a BenchMixer XLQ QuEChERS Vortexer (Benchmark Scientific, Sayreville NJ, U.S.).

#### 2.8.1 UHPLC-MS/MS and data analysis

Chromatographic separation was carried out with a Waters Acquity UPLC H-Class (Waters, Milford, MA) interfaced with a Q-Exactive mass spectrometer (Thermo-Fisher Scientific, Germany) equipped with heated electrospray ionization (HESI) probe. The system was controlled by Xcalibur 3.1 software and was tuned and calibrated using a positive LTQ calibration solution once per week. The LC separation was performed on a Waters Acquity HSS T3 (C18) column (100 × 2.1 mm i. d., 1.8 µm particle size). The mobile phase (0.2 ml/min) consisted of 100% MeCN (solvent A) and 5 mM CH_3_COONH_4_ + 0.1% formic acid in water (solvent B) using positive electrospray ionization. The chromatographic run was completed in 15 min. The elution gradient was as follows: 10% A (0 min), 50% A (5 min), 90% A (12 min), 10% A (14 min). The column temperature was set at 40 °C; the injection volume was 10 μL, and the autosampler temperature was set to 10 °C. The tuning methods and parameters used for all MS acquisitions were optimized under the conditions described below. Spray voltage was set to 3.5 kV (positive); S-Lens RF level, 60; capillary temperature, 350 °C (positive); auxiliary gas temperature, 250°C; nitrogen was used as sheath gas (flow rate 35); auxiliary gas (flow rate 10) and sweep gas (flow rate 2). The acquisition included two different experiment programs: a full-scan (MS1) followed by a DIA (Data Independent Acquisition) experiment to improve the quality of MS2 spectra generated for the target analyte. The MS parameters for the selected method were optimized and described as follows: full MS (35000 K resolution, AGC target 3 × 106, IT 150 ms, scan range 100–500 m*/z*) followed by a DIA experiment to generate MS2 spectra (17500 resolution, AGC 2 × 10^5^, IT auto, Loop count 1, Top N 10, isolation window 1.5 m/z, and NCE 30 eV). Furthermore, an inclusion list with molecular weight and RT window for SER was also included to generate as many micro-scanning events as the precursor ions filtered.

For quantitation of SER in water samples, data were processed using Thermo Xcalibur 3.1 software (Thermo-Fisher Scientific, Germany). Quantification was performed by the internal standard method. The detection limit of the method was 0.05 μg/L. The calibration curve was matrix-matched with fish water in the range of 0.05–200 μg/L. The *R* ^2^ obtained from the calibration curve was 0.9976. The SER precursor (306.0810 m/z) was detected with a retention time (RT) of 6.60 min, with a mass tolerance of 5.0 ppm. The compound was confirmed with its MS2 (158.9757 m/z). Only the adduct ion obtained from the full-scan spectrum was used for the isotopically labeled compound.

### 2.9 Statistical analysis

Data were analyzed with IBM SPSS v25 (Statistical Package 2010; Chicago, IL), and plotted with GraphPad Prism 8.31 for Windows (GraphPad Software Inc, La Jolla, CA) or with Microsoft Excel 2016. Normality was assessed using Kolmogorov-Smirnov and Shapiro-Wilk tests. Multiple comparison tests were used to determine differences between normally distributed groups. One-way ANOVA followed by Tukey’s *post hoc* was applied for groups meeting parametric requirements, whereas the Kruskal–Wallis test followed by Dunn’s multiple comparison test was used for those groups who did not meet parametric assumptions. Water samples were analyzed by pairwise Students’ t-test. Significance was set at *p* < 0.05.

## 3 Results

### 3.1 Experimental concentrations and stability of SER

Nominal concentrations of SER in fish water are presented in Table 3. The lowest concentration, 0.01 μg/L, was below detection limits. The actual concentration corresponding with the nominal concentration of 0.1 μg/L was somewhat higher, this can be because it was not possible to eliminate or effectively compensate for the phenomena of suppression/enhancement of the matrix (assuming using matrix-matched calibration combined with internal standards) due to the high background noise due to its proximity to the detection limit. The remaining experimental concentrations ranged from 102 to 104% of nominal concentrations. SER concentration in fish water following 24 and 48 h remained stable for all conditions except for concentrations 0.1 and 10 μg/L, were it decreased 19.8%–22.0% for 0.1 μg/L, and 12.0%–10.5% for 10 μg/L at 24 and 48 h respectively.

**TABLE 3:** Experimental concentrations of sertraline at 0, 24, and 48 h. Values represent the mean ± SE of three replicates. Results across time (24 and 48 h) were compared to those obtained at time 0 by Student’s t-test, significance was set to *p* < 0.05 and is represented as * when *p* < 0.05. *nd*—not detected.

Nominal Concentration µg/L	Experimental Concentration µg/L at 0 h	Experimental Concentration µg/L at 24 h	Experimental Concentration µg/L at 48 h
0.01	*nd*	*nd*	*nd*
0.1	0.182 ± 0.02	0.146 ± 0.01*	0.141 ± 0.00*
1	1.069 ± 0.06	0.981 ± 0.04	0.927 ± 0.02
10	10.182 ± 0.30	8.959 ± 0.35*	9.118 ± 0.34*

### 3.2 Environmental concentrations of SER did not lead to adverse effects on morphogenesis and growth

**FIGURE 1:**
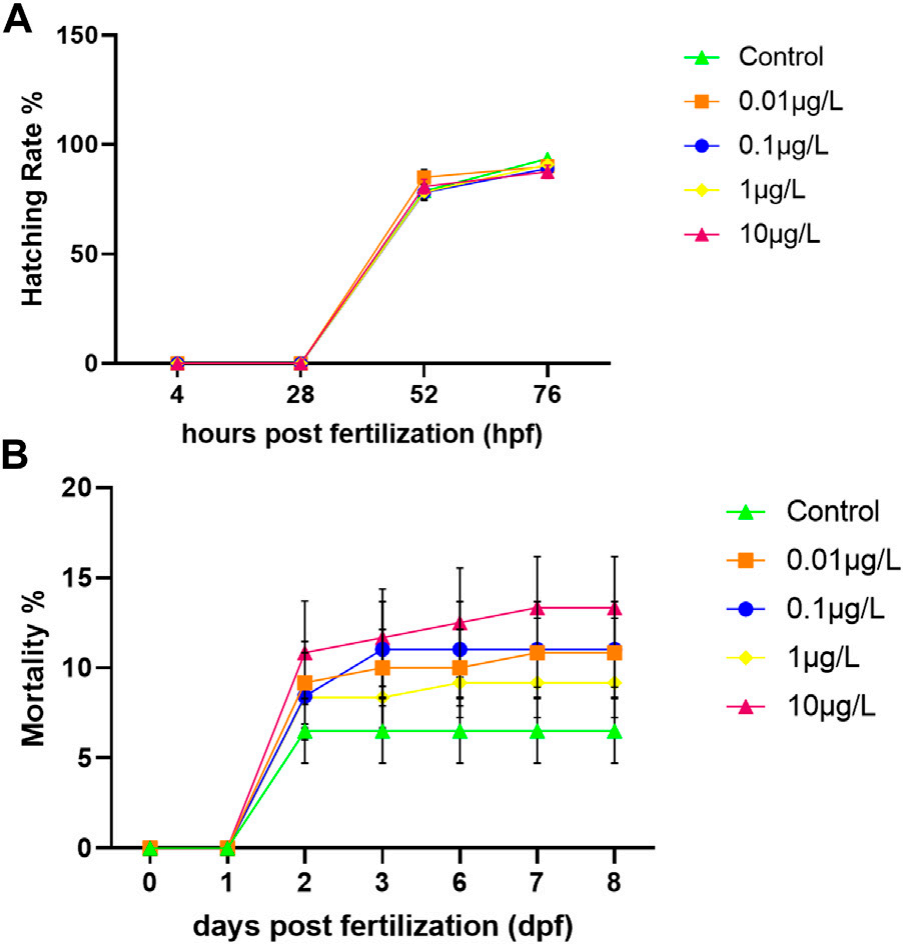
Developmental exposure to different concentrations of Sertraline. **(A)** Hatching rate (%) of control and exposed embryos, recorded daily for 72 h. **(B)** Accumulated mortality of control and exposed embryos/larvae, recorded daily throughout the exposure period. Values represented are mean ± standard error (SE). No statistical differences were observed (*n* = 120) (Tukey post hoc).

**FIGURE 2:**
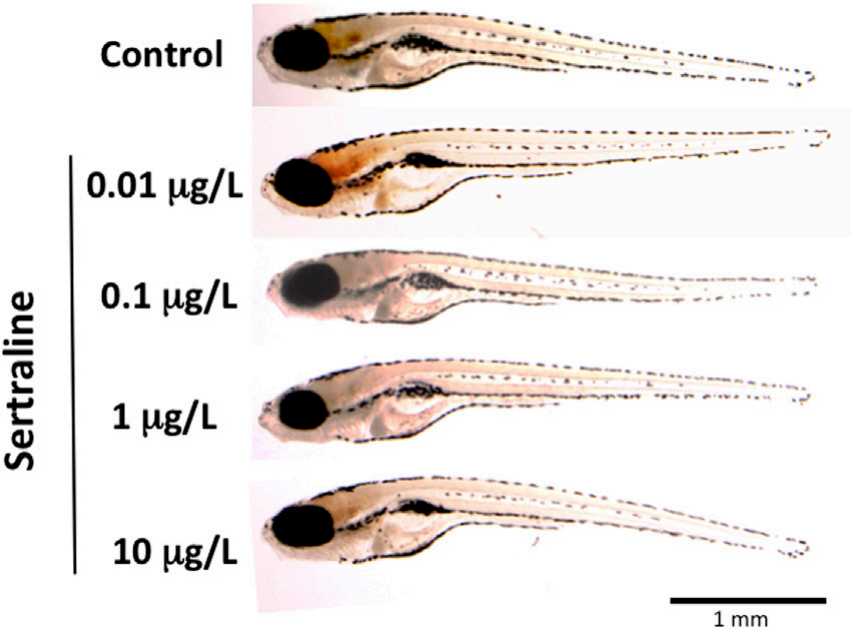
Representative zebrafish larvae (8dpf) following developmental exposure to different concentrations of SER. Lateral view.

Zebrafish embryos were exposed to four environmentally relevant concentrations of SER, from four hpf to eight dpf. Mortality was recorded throughout the whole exposure period and no significance was found for any condition relative to the control (Figure 1B). The lowest accumulated mortality was that of the control registering 6.5 ± 6.2%, while the highest was that of 10 μg/L registering 13.3 ± 9.8% (*p* = 0.121). The hatching rate was also unaffected by SER exposure. In general, at 48 hpf 88% of the surviving embryos had already hatched, reaching 100% at 72 hpf (Figure 1A). At the end of the developmental exposure, zebrafish larvae were fixed and conserved in 90% glycerol for more detailed morphological observations and size measurement. As Figure 2 shows, no changes in larvae morphology was observed across all experimental conditions. Furthermore, larvae’ total length and truck thickness were unaffected by SER developmental exposure (Table 4).

**TABLE 4:** Total length (mm) and trunk thickness (mm) of larvae exposed to different sertraline concentrations during development (4hpf-8dpf). Data are represented as mean ± SE.

Sertraline concentrations µg/L	Total length (mm)	Thickness of Trunk (mm)
control	3.695 ± 0.021	0.198 ± 0.002
0.01	3.721 ± 0.028	0.205 ± 0.003
0.1	3.701 ± 0.039	0.204 ± 0.003
1	3.657 ± 0.015	0.204 ± 0.003
10	3.674 ± 0.039	0.202 ± 0.002

### 3.3 Behavioral responses were altered by developmental exposure to SER

**FIGURE 3:**
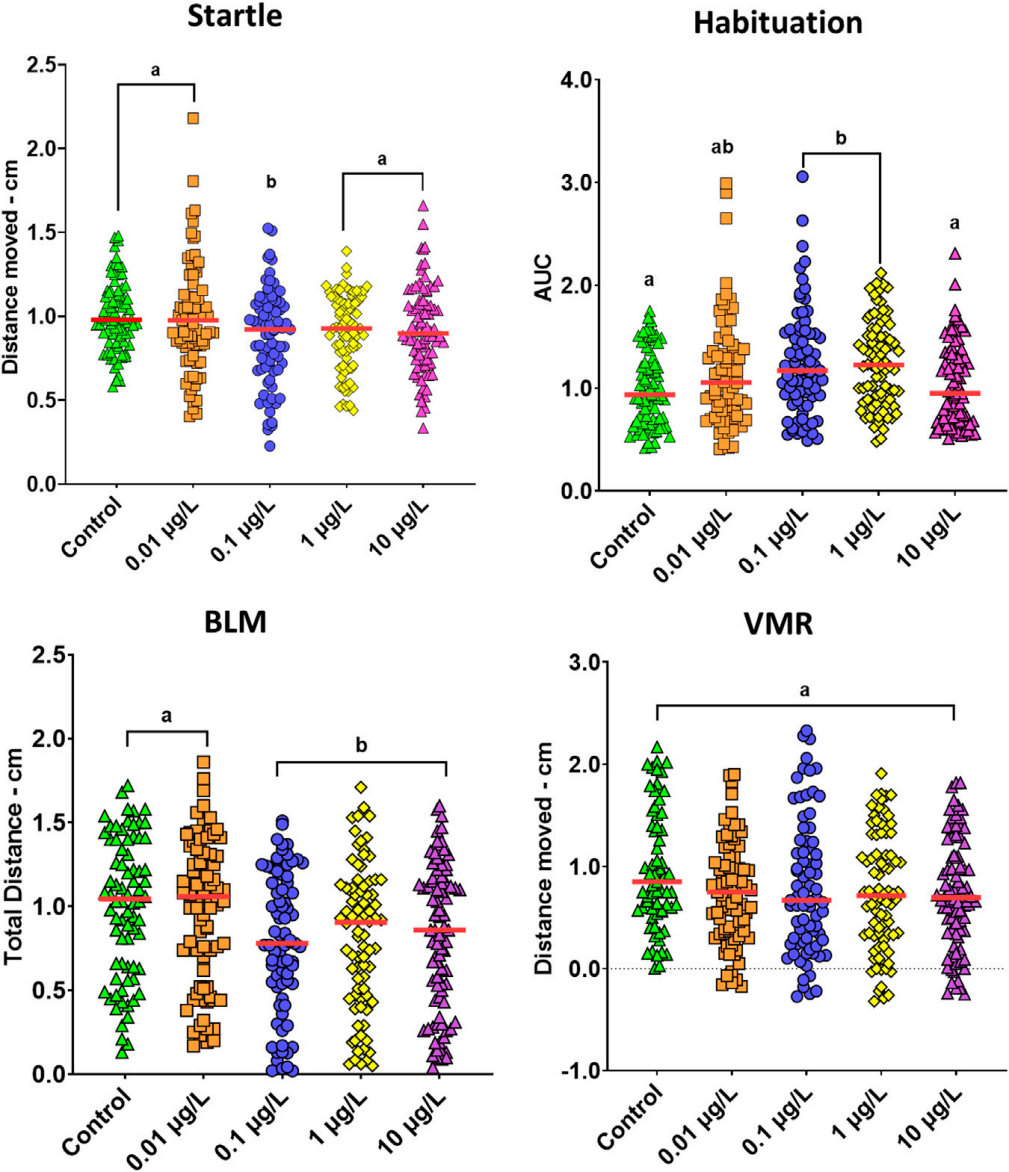
Behavioral changes on zebrafish eight dpf larvae, following developmental exposure (188 h) to different concentrations of SER. Startle - acoustic/vibrational escape response, represented as the total distance (cm) traveled following the delivery of a tapping stimulus (*n* = 79–82); Habituation of the acoustic/vibrational escape response evoked by a series of 50 tapping stimuli delivered every second represented as the area under the curve (AUC) of larvae responses (n = 76–82). Basal Locomotor (BLM) activity, represented as the total distance (cm) traveled during 10min (n = 76–83); Visual-motor response (VMR), reporting the response of larvae due to transition of light to dark, represented as the difference of the total distance (cm) traveled by larvae during 2 minutes after and before the transition of light to dark (*n* = 68–84); Data are from two to three independent experiments and are reported as scatter plots with the median (red line). Different letters indicate statistical subset groups following either Tukey post hoc (Startle and VMR) or Kruskal Wallis test (Habituation and BLM).

Four larvae behaviors were analyzed to assess the effects of early life exposure to SER: 1) the escape response evoked by a vibrational acoustic stimulus, also known as the startle response, 2) habituation to repetitive acoustic stimulation which addresses non-associative learning, 3) basal locomotor activity (BLM), and 4) response to visual stimuli (VMR) (Figure 3). The startle response was the only behavioral parameter that passed the normality test (*p* = 0.160, Shapiro-Wilk) and therefore parametric statistical analysis was applied. One-way ANOVA analysis revealed statistical differences between groups (*F*_(4.397)_ = 2.998, *p* = 0.019). The post hoc test showed a very mild effect in larvae startle, where it was found significantly decreased at only 0.1 μg/L (*p* = 0.045), while the remaining concentrations did not alter this response. Kruskal–Wallis non parametric test divulged significant changes for larvae habituation (*H* (4) = 19.685, *p* = 0.001) and BLM (*H* (4) = 16.457, *p* = 0.002) following SER exposure. A hormetic pattern of response was observed for habituation. A pairwise comparison test showed that the time needed for habituation significantly increased at 0.1 (*p* = 0.004) and 1 μg/L (*p* < 0.001) and then recovered back to control levels at 10 μg/L. On the other hand, larvae showed a significant decrease in locomotor activity from 0.1 μg/L and upwards (*p* < 0.01). Finally, Figure 3 shows that SER does not affect VMR (full plot is available in Supplementary Material S1).

### 3.4 Neurotransmitter profiles and neurotransmitters system genes

**FIGURE 4:**
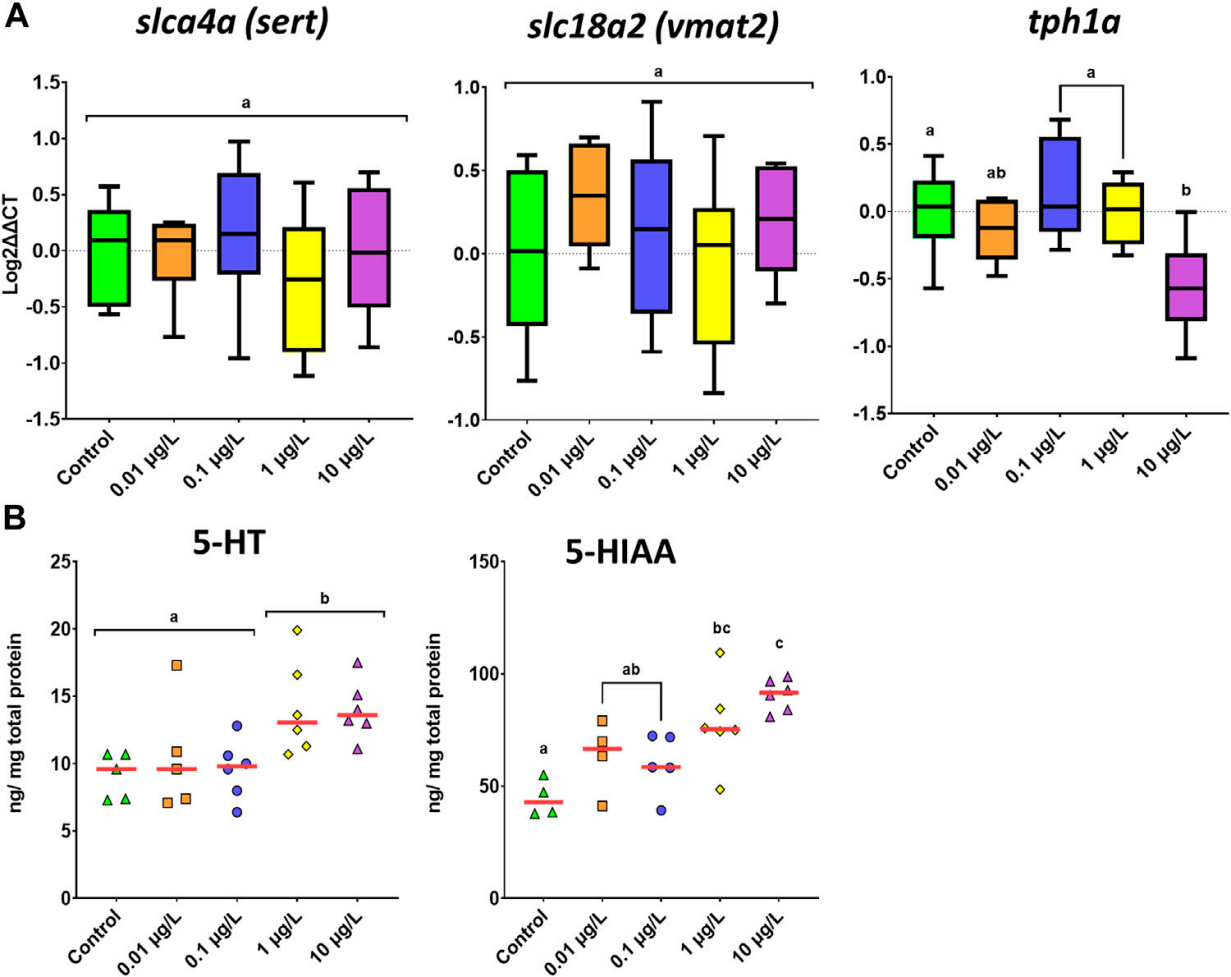
Effects on the serotonergic system of zebrafish larvae following developmental exposure to different concentrations of SER. Data were reported as boxplots or scatter plots (red line—median), for gene expression **(A)** or neurochemical levels **(B)**, respectively. One-way ANOVA followed by Tukey multiple comparison test was used for gene expression (*n* = 6–8), whereas Kruskal Wallis test was used for neurochemical analyses (*n* = 4–6). Different letters represent different subset groups.

**FIGURE 5:**
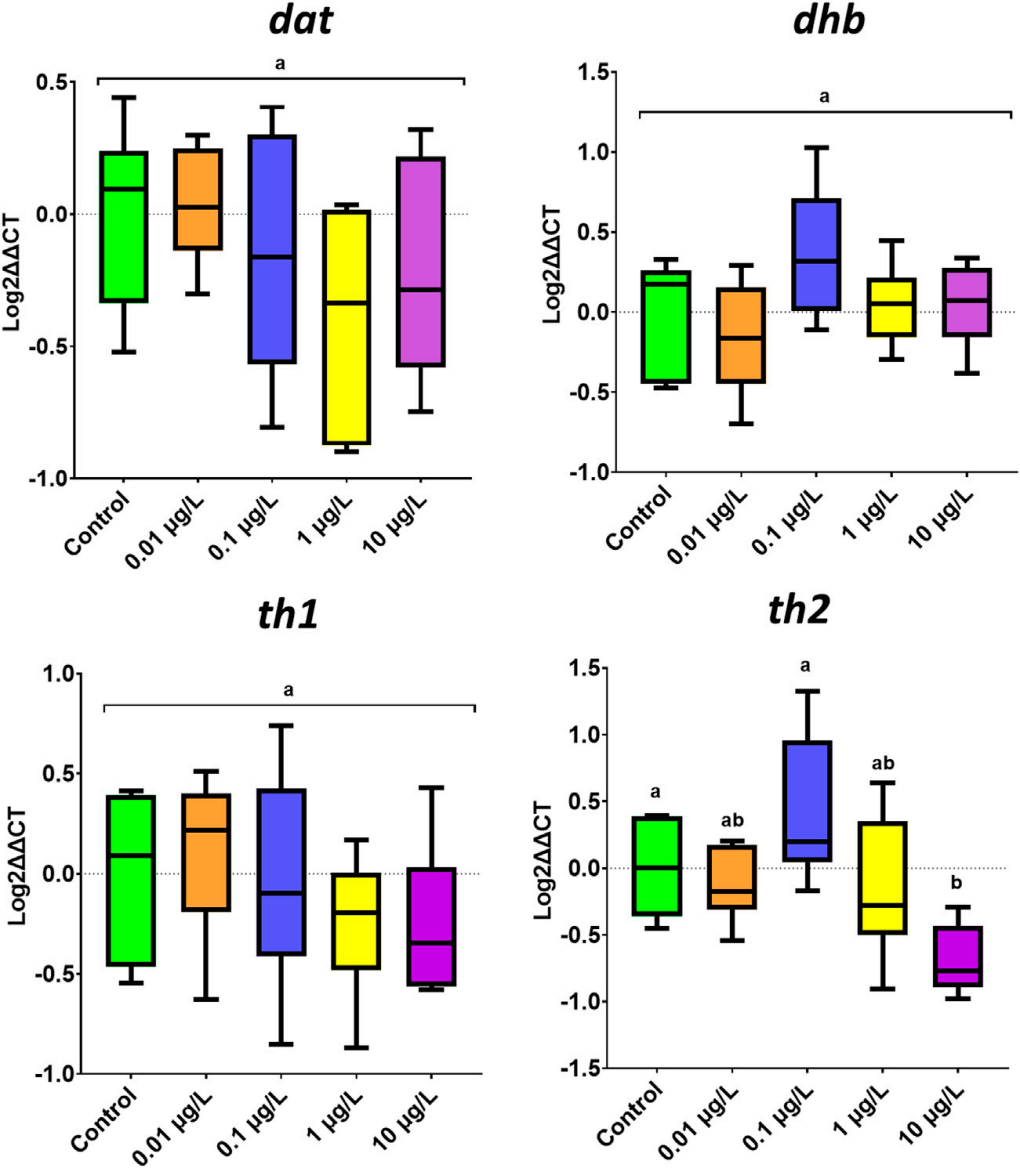
Expression of genes related to the dopaminergic pathway in zebrafish larvae following developmental exposure to SER. Results are represented as boxplots (*n* = 6–8). Different letters represent different subset groups, following Tukey multiple comparison test.

The expression of four genes playing a key role in the serotonergic system was analyzed (Figure 4A). No variation was observed for the reference gene (*ppiaa*) across all treatments (*F*_(4.25)_ = 2.655, *p* = 0.0656, one-way ANOVA). One-way ANOVA analysis showed that only *tph1a* (gene encoding for tryptophan hydroxylase 1a) was significantly affected by developmental exposure to SER (*F*_(4.25)_ = 4.449, *p* = 0.007). The observed difference was mainly due to the downregulation of its expression at the highest exposure concentration (*p* = 0.017). Similarly, SER also showed a very mild effect on the expression of genes related to the dopaminergic system (Figure 5). One-way ANOVA analysis revealed that only *th2* (tyrosine hydroxylase 2) gene gave differences between groups (*F*_(4.24)_ = 5.734, *p* = 0.002), which contributed to the strong downregulation caused by SER 10 μg/L (*p* = 0.038) (Figure 5).

**FIGURE 6:**
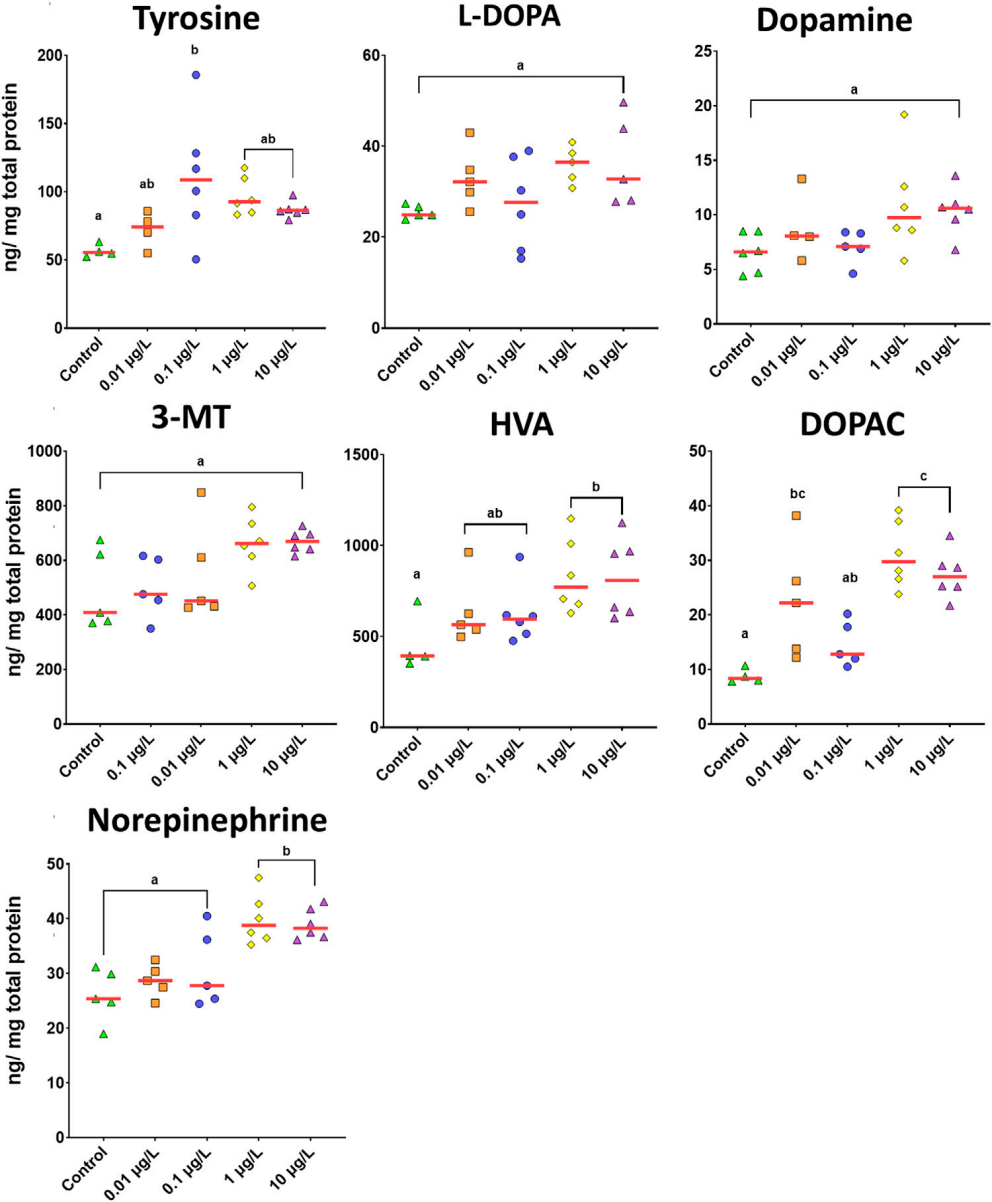
Dopaminergic neurotransmitter profiles of 8dpf zebrafish larvae, following developmental exposure (4hpf to 8dpf) to different concentrations of SER. Results are depicted as scatter plots (*n* = 4–6) with the median (red line) and represented as ng/mg of protein. Different letters represent different subset groups following Tukey post hoc test.

Sertraline’s effect on serotonergic (Figure 4B) and dopaminergic (Figure 6) signaling was then assessed. In general, both pathways were significantly altered due to exposure to the pharmaceutical during early life. For the serotonergic system, levels of both serotonin (5-HT) (F_(4,23)_ = 4.036, *p* = 0.013) and its metabolite (5-HIAA) (F_(4,20)_ = 7.935, *p* = 0.001) increased with SER exposure. These differences were related to the significant increase in 5-HT levels observed in larvae exposed at the two highest concentrations (*p* < 0.030). In addition, an increase of 5-HIAA levels was also dose-dependent, where the highest reported level was reported for the highest concentration of SER (Figure 4B). Interestingly, the major effect of SER on the dopaminergic neurochemical profile was the significant increase in all three metabolites of dopamine: DOPAC (F_(4.21)_ = 10.757, *p* < 0.001), 3-MT (F_(4.21)_ = 3.663, *p* = 0.021) and HVA (F_(4.22)_ = 3.357, *p* = 0.027) (Figure 6). Levels of norepinephrine, a catecholamine synthesized from dopamine, were also found to be increased by SER (F_(4.22)_ = 9.965, *p* < 0.001). The observed increase in dopamine metabolite levels was overall dose-dependent. Effects over DOPAC and 3-MT occurred already at the lowest exposure concentration, especially for DOPAC (*p* = 0.011) (Figure 6). The highest level of 3-MT was reported at 0.1 μg/L (*p* = 0.007) and then remained relatively stable at 1 and 10 μg/L. On the other hand, for DOPAC, the highest reported levels were observed for 1 and 10 μg/L (*p* < 0.001, for both). In the same line as that observed for DOPAC and 3-MT, HVA levels showed a dose-dependent increase, reaching the highest levels at 1 (*p* = 0.019) and 10 μg/L (*p* = 0.022) (Figure 6). Very similar to the above neurochemicals, norepinephrine levels also increased notably at 1 and 10 μg/L (*p* < 0.001, for both). Curiously, despite the observed augmented levels of its transformation products and metabolites, dopamine levels in larvae heads remained unchanged.

## 4 Discussion

Usually, pharmaceutical compounds are designed to target specific proteins, such as transporters or enzymes. SSRIs, such as SER, have been designed to inhibit the reuptake of 5-HT by the serotonin transporter (SERT or SLC6A4), increasing in this way the concentration of 5-HT in serotonergic synapses. The serotonergic system, one of the major neurotransmitter systems, is highly conserved among vertebrate species and responsible for modulating many different behaviors (Valenti et al., 2012). As a consequence of the importance held of this neurochemical, pharmaceuticals designed to affect specific human targets have been reported to affect other vertebrate species, including fish (Winder et al., 2009; Melvin, 2017; Sehonova et al., 2018). However, the physiological after-effects of developmental exposure of aquatic fauna to environmental concentrations of antidepressants are still lacking information. Indeed, SER has been reported to affect zebrafish development. A recent study (Yang et al., 2021) found that embryos exposed to 100 μg/L from ∼6 h to 6 days post-fertilization, showed a 55% acceleration in the hatching rate. However, in this same study, at lower and more relevant concentrations, 1 and 10 μg/L, the authors did not find any developmental impairment. Consistently to Yang et al. report, no developmental effects have been observed in the range of 0.01–10 μg/L SER in our study.

As an SSRI, SER acts on the highly complex serotonergic system which, as previously mentioned, is involved in the regulation of mood and a range of different behaviors as well as in the modulation of neuronal outgrowth (Fricker et al., 2005). Therefore, the altered behavioral activity of zebrafish can be a helpful tool to evaluate developmental neurotoxicity. In this study, a battery of behavioral tests associated with key survival behaviors for fish larvae was analyzed to assess any neurological impairment consequence of developmental exposure to SER. Motor activity in basal conditions (BLM) is an important factor that can determine the optimal navigation of fish in the environment. For example, reduced swimming activity can be associated with reduced foraging and therefore food uptake (Farias et al., 2020). As reported for other SSRIs (Duarte et al., 2020; Farias et al., 2020), SER has been described to decrease zebrafish larvae’ locomotor behavior. For example, Suryanto et al. reported a significant reduction in the swimming activity of 5-day-old larvae following only 24 h of exposure to SER concentrations ranging from 10 μg/L to 1 mg/L (Suryanto et al., 2021). Other studies also reported a significant decrease in either larva mobile cumulative duration or the number of spontaneous swimming bouts, respectively, following developmental exposure to either 1, 10, or 100 μg/L of SER (Huang et al., 2019; Yang et al., 2021). In agreement with these studies, we also observed that SER exposure led to an important decrease in the total distance traveled monitored for 10 min under dark conditions. Furthermore, this decrease was observed at concentrations as low as 0.1 μg/L, which is in the range of SER concentrations found in surface waters (Mole and Brooks, 2019). Fish larvae survival is limited by multiple factors and to survive, larvae must be able to quickly respond and swim away from any threat, which is defined as the startle response. Any deviation in the normal physiological process of this response may have adverse consequences. Despite the observed diminished locomotor activity, in SER exposures ≥0.1 μg/L, larvae startle response was only mildly weakened by this compound. Other SSRIs have been reported to affect this response under different exposure scenarios (Painter et al., 2009; Faria et al., 2021). However, it is the study conducted by Painter et al., 2009, which has a similar exposure scenario to ours, but with fathead minnows (*Pimephales promelas*), that found no changes in the startle response, which is to some extent, comparable with our observations for zebrafish. Habituation is a non-associative type of learning which allows individuals to navigate through a multi-stimulus environment with as less energy cost as possible by filtering non-threatening stimuli, and therefore rapidly diminishing any unnecessary responses. To our knowledge, the effects on the habituation of zebrafish larvae after being reared in the presence of SER, have not yet been explored. In this study, we found that response patterns of larvae to consecutive acoustic/vibrational stimuli displayed a non-monotonic pattern (hormetic effect), presenting therefore a biphasic curve. Hormetic effect is a dose-response phenomenon that refers to a biphasic or multiphasic response to a drug or toxin. Hormetic effects are commonly triggered when a drug interacts with homeostatic mechanisms, and has been found in other studies of fish exposed to sertraline or other (Maximino et al., 2011; Xie et al., 2015). However, the mechanisms underlying this phenomenon are still unclear. At concentrations ≤1 μg/L, larvae were slow to learn the harmless nature of the delivered stimulus, therefore showing a larger AUC, whereas, at the highest concentration, this effect was reestablished back to control levels. Disruption of zebrafish larvae habituation in the opposite direction was observed by another SSRI, fluoxetine, in a previous study conducted by our group (Faria et al., 2021). Specific effects of each drug, potential differences in the response with exposure time or age-dependent effects might be behind the observed differences between our results with sertraline (188 h-exposure; 4–192 hpf) and the fluoxetine reports (24 h-exposure; 168–192 hpf). Finally, we aimed to assess if SER could disrupt larvae responses to light stimulus, termed the visual motor response (VMR). This behavior, reflects the anxiety and stress behavior of larvae towards sudden shifts of light, in particular, the sudden absence of light, triggering an abrupt increase in swimming activity that can last 10–15 min (Burton et al., 2017). Interestingly, SER was unable to alter larvae response to this stimulus at the tested concentrations, despite having decreased larvae locomotor activity. Curiously, this response was similar to that observed by Huang et al., who, in developmentally exposed zebrafish larvae, saw a decrease in the number of swimming movements for SER 1 and 10 μg/L and the absence of VMR (Huang et al., 2019). In the same study, the VMR was only seen altered by SER at the highest tested concentration of 100 μg/L, suggesting that relevant concentrations of SER do not alter larvae visual response.

As an SSRI, SER is an inhibitor of the serotonin (SERT) transporter, thus blocking the reuptake of serotonin from the synaptic cleft into the presynaptic neuron. Furthermore, although not considered the primary mechanism by which SER and other SSRIs exert their therapeutic effect, it is also known to have a minor inhibitor effect over the dopamine and norepinephrine (noradrenaline) transporters (Comer and Figgitt, 2000). As the monoaminergic system is one of the main modulators of locomotor activity, we aimed to further explore whether the behavioral abnormalities could be explained by the influences on these neurotransmitter profiles. As such, we found that levels of both serotonin and its metabolite (5-HIAA) increased in larvae heads at 1 and 10 μg/L of exposure. Interestingly, it seems that 5-HT levels remained high despite the increase in its metabolism. The changes in neurochemical levels are only slightly reflected at the molecular level, as on the one hand, neither transporter gene showed altered expressions and, on the other hand, the down-regulation of *tph1a*, a gene encoding for the enzyme tryptophan hydroxylase, responsible for 5-HT synthesis, could be a feedback response to the increased levels of 5-HT. Curiously, larvae mainly showed significant hypo-locomotion and slower habituation at ≥0.1 μg/L, which could be associated with the sedative effects driven by increased levels of serotonin. Contrarily to this study, Yang et al., despite finding a decrease in larvae locomotion, did not find altered serotonin levels in zebrafish larvae following developmental exposure to similar and higher SER concentrations. On the other hand, this same study found upregulation of the transporter gene *serta*, especially at 10 μg/L (Yang et al., 2021). The discrepancy in these results could be confounded by slight modifications of the exposure conditions, such as the time of exposure since in this study organisms were exposed for two more days, as well as of medium characteristics. For example, it has been proven that higher pH levels allow SER to stay in its non-ionized form and therefore more readily cross cell membranes (Alsop and Wilson, 2019). In this regard, in our study, the pH of the medium was 0.6 points higher than that of Yang et al., 2021.

Finally, SER also altered normal dopamine and norepinephrine pathways. Dopamine levels in exposed organisms remained unchanged, despite the down-regulation of *th2* gene, which encodes for the enzyme tyrosine hydroxylase, which catalyzes the rate-limiting step in the synthesis of catecholamines. Dopamine is a key neuromodulator involved in the control of motor systems in both invertebrates and vertebrates (Schotland et al., 1995; Crisp and Mesce, 2004) and it is known that impairment of the dopaminergic system leads to modified locomotion in teleosts (Irons et al., 2013; Tran et al., 2015). Curiously, despite the unchanged dopamine levels, dopamine metabolism was increased by SER, which suggests that one cannot discharge an off-target effect of developmental exposure to SER on catecholamines. Indeed, previous studies have described that SER has a moderate affinity for dopamine transporters (Sánchez and Hyttel, 1999) and has the ability of dopamine reuptake inhibition (Goodnick and Goldstein, 1998), however, in this study the neurodevelopmental significance of its actions over this system is unclear. Norepinephrine is synthesized from dopamine by dopamine β-monooxygenase (DBH) and is responsible for preparing the brain and body for action, such as the fight-or-flight response, which is essentially the larvae’s startle/escape response. Levels of norepinephrine were significantly high for concentrations 1 and 10 μg/L, despite the lack of upregulation of gene expression that encodes for DBH. Nevertheless, the increase in norepinephrine levels was not reflected in larvae behavior, since the startle response was weakly affected and in the opposite direction to that expected for higher norepinephrine levels. These results suggest that while serotonin was predominantly involved in our observed behavioral changes, we have produced evidence that developmental exposure to sertraline can impact the catecholamine system.

## 5 Conclusion

This study allowed us to improve our understanding of the behavioral effects of sertraline in a developmental concept. We found that fish exposure to environmentally relevant concentrations of sertraline during early developmental stages suggests that behaviors that allow fish to navigate through the wild are altered. Sertraline reduced and delayed fish locomotion and learning respectively, being the decrease in fish locomotion being the most persistent behavioral response in all studies. Finally, changes in serotonin levels were more supportive of behavioral dysfunction performance than other neurochemicals and molecular markers, emphasizing the relationship between serotonin signaling and behaviors in zebrafish. In future studies, it would be interesting to include ecologically relevant behaviors such as predator avoidance and feeding to better translate laboratory studies to the environment.

## Data Availability

The raw data supporting the conclusion of this article will be made available by the authors, without undue reservation.
