# Supplementary material for: Developmental exposure to sertraline impaired zebrafish behavioral and neurochemical profiles
Source: Front Physiol. 2022 Nov 18;13:1040598. doi: 10.3389/fphys.2022.1040598 (PMC9716079; doi:10.3389/fphys.2022.1040598)
Supplement: Supplementary file 1 [file Table1.DOCX]

**Supplementary Material**

**Developmental exposure to sertraline impaired zebrafish behavioral and neurochemical profiles**

**Melissa Faria^1*^, Marina Bellot^2,^, Oscar Soto^3^, Eva Prats^4^, Nicola Montemurro^1^, Diana Manjarrés^1^, Cristian Gómez-Canela^2^, Demetrio Raldúa^1^**

^1^Institute for Environmental Assessment and Water Research (IDAEA-CSIC). Jordi Girona 18, 08034 Barcelona, Spain

^2^Department of Analytical and Applied Chemistry (Chromatography section), School of Engineering, Institut Químic de Sarrià-Universitat Ramon Llull, Via Augusta 390, 08017 Barcelona, Spain.

^3^ Universitat Ramon Llull, Via Augusta 390, 08017 Barcelona, Spain.

**Results**

**
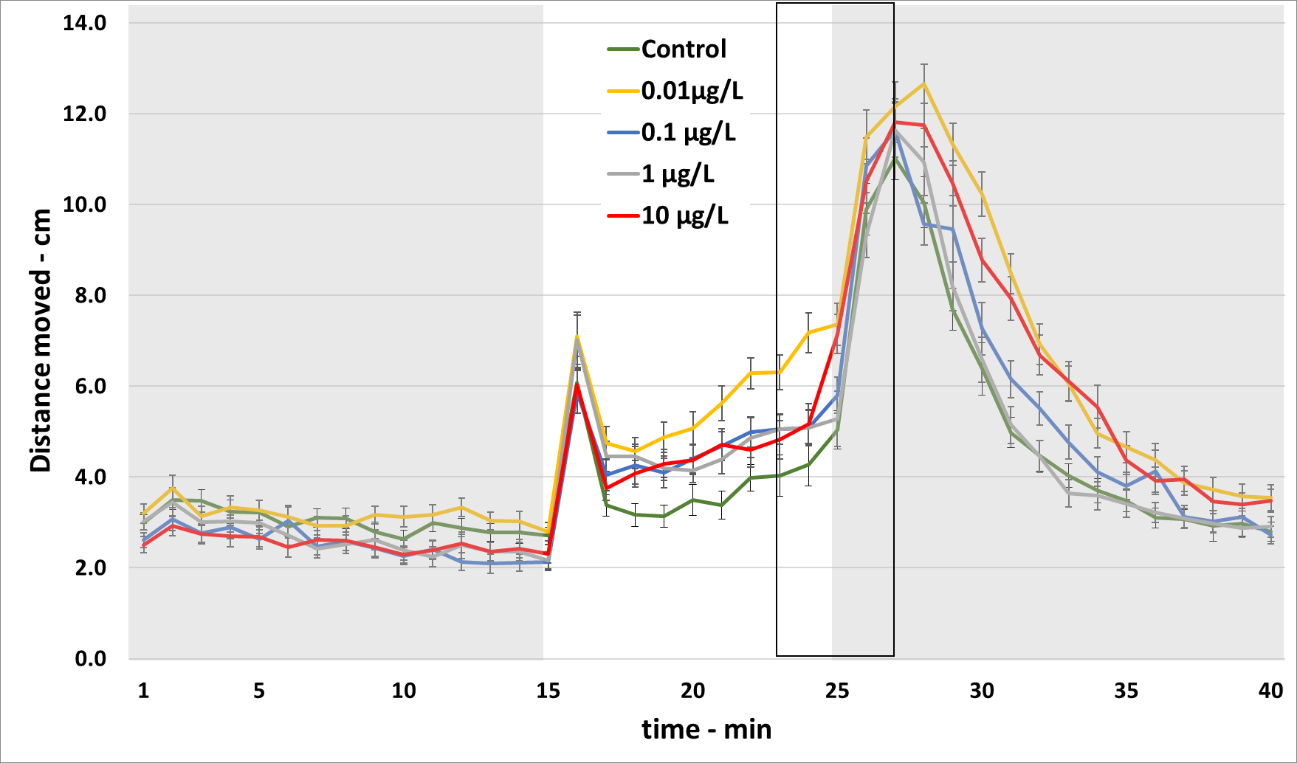
**

**Supplementary Figure 1** – Plotted visual motor responses (VMR) of control and exposed zebrafish larvae. Video tracking conditions consisted of a 40 min cycle including a 15 min dark period (first shaded rectangle), followed by a 10 min light period and then a second 15 min dark cycle (second shaded rectangle). The VMR reports larvae responses following a transition of light to dark and is represented as the difference of the total distance (cm) traveled during two minutes after and before the transition (n=64-84) (black lined rectangle).
